# SARS-CoV-2 NSP5 and N protein counteract the RIG-I signaling pathway by suppressing the formation of stress granules

**DOI:** 10.1038/s41392-022-00878-3

**Published:** 2022-01-24

**Authors:** Yi Zheng, Jian Deng, Lulu Han, Meng-Wei Zhuang, Yanwen Xu, Jing Zhang, Mei-Ling Nan, Yang Xiao, Peng Zhan, Xinyong Liu, Chengjiang Gao, Pei-Hui Wang

**Affiliations:** 1 Key Laboratory of Infection and Immunity of Shandong Province, Department of Immunology, School of Basic Medical Sciences, Cheeloo College of Medicine, Shandong University, Jinan, 250012 China; 2 Key Laboratory for Experimental Teratology of Ministry of Education and Advanced Medical Research Institute, Cheeloo College of Medicine, Shandong University, Jinan, 250012 China; 3 Department of Medicinal Chemistry, Key Laboratory of Chemical Biology (Ministry of Education), School of Pharmaceutical Sciences, Cheeloo College of Medicine, Shandong University, 44 West Culture Road, 250012 Jinan, Shandong PR China; 4 China–Belgium Collaborative Research Center for Innovative Antiviral Drugs of Shandong Province, 44 West Culture Road, 250012 Jinan, Shandong PR China; 5 Suzhou Research Institute, Shandong University, Shandong University, Suzhou, Jiangsu 215123 China

**Keywords:** Innate immunity, Infectious diseases

## Abstract

As a highly pathogenic human coronavirus, SARS-CoV-2 has to counteract an intricate network of antiviral host responses to establish infection and spread. The nucleic acid-induced stress response is an essential component of antiviral defense and is closely related to antiviral innate immunity. However, whether SARS-CoV-2 regulates the stress response pathway to achieve immune evasion remains elusive. In this study, SARS-CoV-2 NSP5 and N protein were found to attenuate antiviral stress granule (avSG) formation. Moreover, NSP5 and N suppressed IFN expression induced by infection of Sendai virus or transfection of a synthetic mimic of dsRNA, poly (I:C), inhibiting TBK1 and IRF3 phosphorylation, and restraining the nuclear translocalization of IRF3. Furthermore, HEK293T cells with ectopic expression of NSP5 or N protein were less resistant to vesicular stomatitis virus infection. Mechanistically, NSP5 suppressed avSG formation and disrupted RIG-I–MAVS complex to attenuate the RIG-I–mediated antiviral immunity. In contrast to the multiple targets of NSP5, the N protein specifically targeted cofactors upstream of RIG-I. The N protein interacted with G3BP1 to prevent avSG formation and to keep the cofactors G3BP1 and PACT from activating RIG-I. Additionally, the N protein also affected the recognition of dsRNA by RIG-I. This study revealed the intimate correlation between SARS-CoV-2, the stress response, and innate antiviral immunity, shedding light on the pathogenic mechanism of COVID-19.

## Introduction

Innate antiviral immunity plays an essential role in the initial detection and control of the spread of viral infection. Nucleic acids produced by viruses in infected cells can be recognized by the pattern recognition receptors (PRRs), leading to the activation of innate immune signaling cascades.^1^ Viral nucleic acids including DNA and RNA are detected by distinct PRRs. Cyclic GMP-AMP synthase (cGAS) senses double-stranded DNA (dsDNA) and triggers the production of the second messenger cGAMP. Then, cGAMP binds to the downstream endoplasmic reticulum (ER) adaptor molecule STING.^2^ RIG-I–like receptors (RLRs), including RIG-I and MDA5, recognize viral dsRNA and activate the mitochondrial adaptor molecule MAVS.^3^ The cGAS-STING and RIG-I/MDA5 signaling pathways ultimately converge on TBK1, the kinase for the transcription factor IRF3. Phosphorylation of IRF3 facilitates its dimerization and nuclear translocation to initiate transcription of type I and type III interferons (IFNs), which will activate the JAK-STAT pathway to induce the production of interferon-stimulated genes (ISGs) and establish an antiviral state.^1^

Stress granules (SGs), induced by various types of stress, are the cytoplasmic aggregates of mRNA- and RNA-binding proteins. These different types of stress, such as oxygen, heat shock, and nucleic acids, result in the phosphorylation of EIF2α.^4^ The phosphorylation of EIF2α is catalyzed by protein kinase R (PKR) following the sensing of nucleic acids, which leads to a concomitant mRNA translation shutoff.^5^ The stalled translation complex then aggregates with SG hub proteins, such as Ras GTPase-activating protein-binding protein 1 (G3BP1) and T cell-restricted intracellular antigen 1 (TIA-1), to form SGs, leading to the storage of translation factors, RNA-binding proteins, and signaling molecules.^4^ Nucleic acids produced from a viral infection can activate PKR and its downstream signaling cascade to stimulate the formation of SGs. However, an ample number of viruses from different families encode SG antagonists, such as NS1 of IAV and p4a of MERS, to block the formation of SGs and reorganize the cellular environment to optimize viral infection.^6,7^ Some viruses also employ SG components in the viral replication compartment to enhance viral infection. Recently, SGs have been considered as an essential platform for the activation of cGAS-STING and RIG-I/MDA5 signaling pathways. G3BP1, the nucleating protein of SGs, interacts with RIG-I and promotes its activation.^8^ In addition, G3BP1 associates with cGAS and enhances the oligomerization and DNA binding activity of cGAS.^9^ In addition to G3BP1, PKR is critical for both cGAS-STING and RIG-I/MDA5 signaling pathways.^10^ These phenomena suggest that the two pathways are closely connected and can counteract viral infections.

COVID-19 is an infectious disease caused by SARS-CoV-2 infection.^11,12^ In COVID-19 patients, antiviral immunity is suppressed, but proinflammatory responses are evoked, suggesting that SARS-CoV-2 efficiently modulates the host immune response. After viral entry, the positive RNA genome of SARS-CoV-2 can be translated into a long polypeptide, which is then digested by two viral proteases, NSP3 (PLpro) and NSP5 (3Cpro).^11^ NSP5 is the main protease (Mpro) of the SARS-CoV-2. NSP5 also cleaves NLRP12 and TAB1 in addition to processing long viral polypeptides,^13^ suggesting that the protease activity of NSP5 is essential for both viral infection and reorganization of the cellular environment. Since it is an enzyme that is critical for viral replication and its cleavage specificity is very distinct from the known human proteases, NSP5 is an attractive drug target for treating COVID-19.^14^ As the most abundant viral protein during SARS-CoV-2 infection, the N protein contains the N-terminal domain (NTD) and the C-terminal domain (CTD), which are primarily responsible for RNA binding and critical for viral genome assembly into the virions.^15^ Therefore, the N protein is another appealing antigen and drug target. Both NSP5 and N play an indispensable role in viral proliferation, but whether they modulate the host cell environment to facilitate viral replication is not well studied.

Here, we reported that the NSP5 and N proteins of SARS-CoV-2 attenuated antiviral SG (avSG) formation and dampened the RIG-I/MDA5-mediated type I and type III IFN response. Consequently, the overexpression of NSP5 or N protein promoted virus replication. Mechanistically, NSP5 diminished avSG formation independent of its protease activity. Moreover, NSP5 directly targeted RIG-I and impeded RIG-I–MAVS interaction to affect downstream signaling. In contrast to NSP5, the N protein interacted with G3BP1, sequestered G3BP1 to block SG formation, and prevented the cofactors of RLRs from enhancing the activity of RIG-I, thus, affecting dsRNA sensing. This study uncovered the close association between SARS-CoV-2, the stress response, and innate antiviral immunity, thus providing insights into viral pathogenicity and treatment.

## Results

### SARS-CoV-2 NSP5 and N protein inhibit avSG formation

**Fig. 1:**
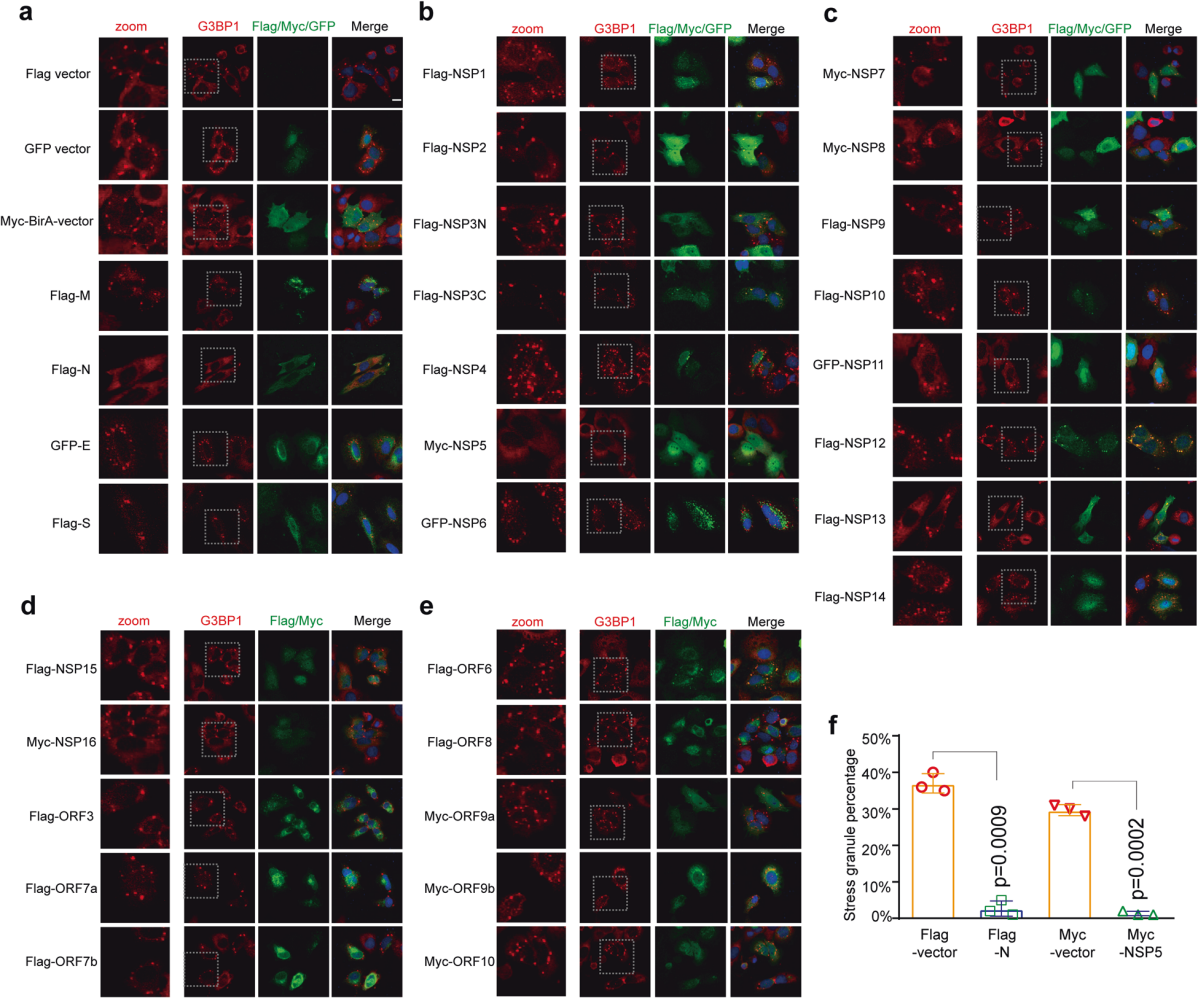
SARS-CoV-2 NSP5 and N inhibit avSG formation. **a**–**e** HeLa cells seeded on coverslips in 12-well plates (5 × 10^4^ cells per well) were transfected with an empty vector as a control or a plasmid expressing each viral protein as indicated. Each plasmid was transfected with 1.5 µg per well. Twenty-four hours after transfection, the cells were subjected to fixation, permeabilization, and blocking as described in the Methods section. The cells were further stained with antibodies as indicated. Scale bar, 10 μm. **f** The percentage of cells with SGs was quantified in the *N* and NSP5 groups and their corresponding control groups (*N* = 200)

MERS-CoV and infectious bronchitis virus are equipped with viral antagonists to counteract the formation of avSGs,^7,16^ indicating that this is likely a conserved evasion strategy for coronaviruses. We hypothesize that SARS-CoV-2 likely modulates avSG formation to support viral replication. To investigate which viral protein is involved in regulating this process, we transfected plasmids encoding each of the SARS-CoV-2 proteins into HeLa cells. Then, we examined SG formation by staining for the SG marker protein G3BP1 together with the tagged viral proteins. A previous study suggested that this is a convenient and versatile methodology that led to the discovery that p4a of MERS-CoV is an SG antagonist.^7^ This suggests that it is a reliable method to examine the effects of particular proteins on the formation of SGs, since the eukaryotic plasmid transfection leads to the formation of SGs in a PKR-dependent manner.^7^ Similar to the previous findings, we observed that the control vectors, pCAG-Flag, pcDNA6B-BirA-Myc, and pEGFP, efficiently induced SG formation in approximately 30% of the transfected cells (Figs. 1a, f). Furthermore, we observed that the N and NSP5 proteins decreased the percentage of cells with SGs to less than 5% (Figs. 1a, b, f), exhibiting a more potent capability to counteract avSG formation than the other proteins, including structural and nonstructural proteins (Fig. 1a-e). Collectively, we found that SARS-CoV-2 encodes at least two proteins, N and NSP5, that regulate avSG formation resulting from plasmid transfection.

### SARS-CoV-2 NSP5 and N protein promote viral replication

**Fig. 2:**
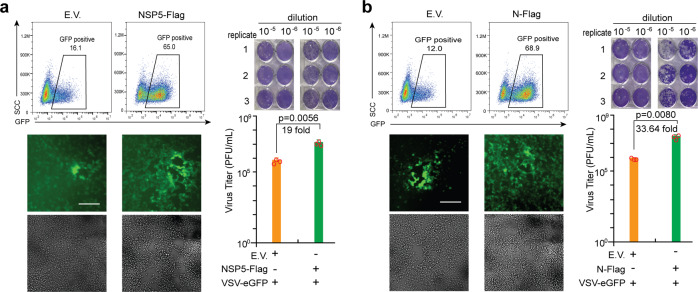
SARS-CoV-2 NSP5 and N facilitate viral replication. The HEK293T cells were subjected to transfection with empty vector (E.V.), NSP5 (**a**)-, or N (**b**)-expressing plasmids as indicated for 24 h. The cells were subsequently infected with VSV-eGFP (MOI = 0.001) for 12 h before imaging and flow cytometry analysis. The culture supernatant collected at 20 h postinfection was used to determine viral titers (PFU per mL) via plaque assays. The fluorescent imaging and flow cytometry data are representative of two independently performed experiments with similar results. Scale bar, 50 μm. In plaque assays, data are presented as mean values ± SEM from triplicate infections from one representative experiment of two. Statistical significance is shown as indicated. EV empty vector, h hours, PFU plaque-forming units.

Given that SGs formation is one of the innate defense systems responsible for translation inhibition and restricting viral infection,^5^ we investigated whether NSP5 and N protein played any role in regulating viral replication. VSV-eGFP was chosen as the model virus to examine the effect of N protein and NSP5 on viral infection and replication due to the lack of a biosafety level three laboratory. We observed that VSV-eGFP replication was significantly facilitated in HEK293T cells transfected with NSP5-expression plasmids compared with those transfected with empty vector via both flow cytometry and fluorescence microscopy analysis (Fig. 2a, left panel). Consistently, we observed more plaques by the titration assay from the cultured supernatant of HEK293T cells expressing NSP5 than that from the supernatant of HEK293T cells transfected with an empty vector (Fig. 2a, right panel). Similar to the NSP5, HEK293T cells expressing N protein were more susceptible to VSV-eGFP infection and replication (Fig. 2b). In summary, these evidences suggested that the NSP5 and N protein promoted viral replication, likely by inhibiting SG formation.

### SARS-CoV-2 NSP5 and N protein inhibit IFN induction

**Fig. 3:**
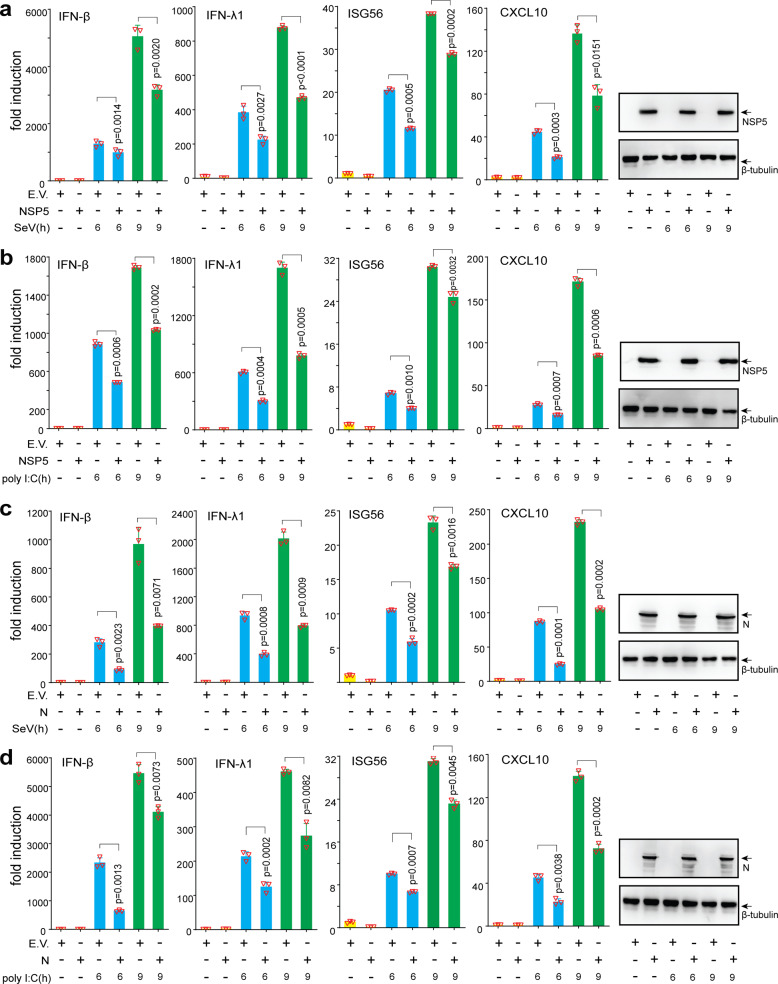
NSP5 and N proteins inhibit IFN production. A549 cells were transfected with plasmids of empty vector, NSP5 (**a** and **b**), or N protein (**c** and **d**) as indicated, 24 h later. the cells were further stimulated with SeV (**a** and **c**) or poly (I:C) (**b** and **d**). At the indicated time points, cells were collected for RT-qPCR analysis to determine the relative expression levels of target genes normalized by GAPDH. Data are presented as mean values ± SEM from three biological replicates from one representative experiment of two. EV empty vector, h hours.

In addition to shutting off translation to suppress viral replication, SGs serve as platforms for the RLR-mediated IFN response.^8^ Multiple PRRs and intermediate signaling molecules, including RIG-I, TRIM25, and PKR-activating protein (PACT), are present in avSGs during viral infection or nucleic acid transfection,^16^ and SG marker proteins are crucial for the RLR- and cGAS-STING-mediated IFN response.^10^ Since NSP5 and N proteins affect SG formation, we hypothesized that both proteins may negatively regulate the type I and type III IFN response. To investigate whether SARS-CoV-2 NSP5 or N protein affects innate antiviral immunity, A549 and HEK293T cells expressing NSP5 or N protein were subjected to infection with Sendai virus (SeV) or transfection with dsRNA mimic poly (I:C). We observed that the expression of IFN-β, IFN-λ1, and the two ISGs, ISG56 and CXCL10, was effectively stimulated by both SeV infection and poly (I:C) transfection. However, the induced expression of those genes was significantly decreased in A549 and HEK293T cells expressing NSP5 (Fig. 3a, b; supplementary Fig. S1) or N protein (Fig. 3c and d; supplementary Fig. S1). Collectively, SARS-CoV-2 NSP5 and N protein inhibited SeV- and poly (I:C)-induced expression of IFN-β, IFN-λ1, ISG56 and CXCL10.

### SARS-CoV-2 NSP5 functions in multiple steps of the RLR signaling pathway

**Fig. 4:**
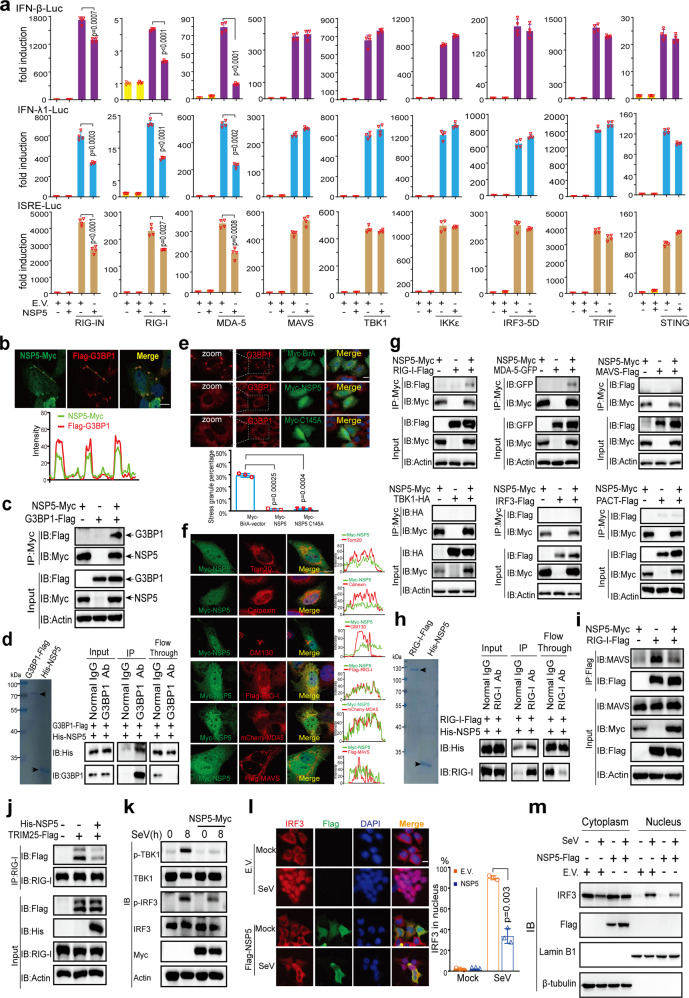
NSP5 inhibits RLR-induced IFN activation. **a** IFN-β, IFN-λ1, or ISRE luciferase reporters and protein-expressing plasmids were transfected into HEK293T cells as indicated. pRL-TK plasmid was transfected to normalize transfection efficiency. After transfection for 36 h, the cells were lysed to examine the luciferase activities by dual-luciferase assays. **b** NSP5 colocalizes with SG marker G3BP1. Top panel: Representative confocal images of NSP5-Myc colocalization with G3BP1-Flag in HeLa cells. Scale bar, 10 µm. Bottom panel: Line profiling of NSP5-Myc with G3BP1-Flag. The intensity of each line was measured by ImageJ software and drawn by GraphPad Prism 8.0. **c** Co-IP analysis of the interaction between NSP5-Myc and G3BP1-Flag in HEK293T cells. HEK293T cells were transfected with the indicated plasmids for 24 h before co-IP with Myc antibody. Results shown are representative of two independent experiments. **d** NSP5 directly binds to G3BP1. Left panel: Coomassie blue staining analysis of the purified G3BP1-Flag and His-NSP5 proteins. Right panel: Co-IP analysis of the in vitro interaction between G3BP1-Flag and His-NSP5. **e** NSP5 inhibits SG formation induced by plasmid transfection. Top panel: Confocal microscopic analysis of SG formation in HeLa cells transfected with plasmids of Myc-BirA (control group), Myc-NSP5, or Myc-NSP5 C145A for 24 h. Scale bars, 10 μm. Bottom panel: Quantification analysis of the percentage of SG formation (50 cells per sample). **f** NSP5 colocalizes with RIG-I and MDA5. Representative confocal images of immunofluorescence staining for NSP5-Myc with the indicated organelles or signaling molecules in HeLa cells. Scale bar, 10 µm. Tom20, mitochondrial marker; Calnexin, ER marker; GM130, Golgi marker. **g-i** NSP5 interacts with RLRs and prevents the interaction of RIG-I and MAVS. **g** NSP5 interacts with RIG-I and MDA5 but not with MAVS, TBK1, IRF3, or PACT. HEK293T cells were transfected with the indicated plasmids for 24 h before coimmunoprecipitation. The input and immunoprecipitates were immunoblotted with the indicated antibodies. The pcDNA6B vector was used to balance the total amount of DNA in each transfection. Immunoblotting results are representative of two independent experiments. **h** NSP5 directly binds to RIG-I. Left panel: Coomassie blue staining analysis of the purified RIG-I-Flag and His-NSP5 proteins. Right panel: Co-IP analysis of the in vitro interaction between RIG-I-Flag and His-NSP5. **i** and **j** NSP5 impairs the RIG-I–MAVS and RIG-I–TRIM25 interactions. HEK293T cells were transfected with the indicated plasmids for 24 h. Coimmunoprecipitation and immunoblot analyses were performed with the indicated antibodies. **k** NSP5 inhibits the phosphorylation of TBK1 and IRF3 induced by SeV infection. HeLa cells were transfected with NSP5-expressing plasmids and subsequently infected with SeV as indicated. The expression of total and phosphorylated (p-) TBK1, total and phosphorylated (p-) IRF3, and NSP5 was detected by immunoblotting. **l** and **m** NSP5 prevents the nuclear translocation of IRF3. **l** Left panel: Confocal microscopic analysis of IRF3 localization in HEK293T cells transfected with an empty vector or NSP5-expressing plasmid for 24 h, followed by SeV infection. Scale bars, 10 μm. Right panel: Quantification analysis of IRF3 nuclear localization (50 cells per sample). **m** Immunoblot analysis of cytoplasmic and nuclear IRF3. HEK293T cells were transfected with an empty vector or NSP5-expression plasmid. Twenty-four hours later, the cells were infected with SeV for 6 h and the cytoplasmic and nuclear proteins were fractionated. The fractions were immunoblotted with antibodies of IRF3, NSP5, Lamin B1 (nuclear marker), and β-tubulin (cytoplasmic marker). EV empty vector, h hours.

We next studied the mechanism by which SARS-CoV-2 NSP5 antagonizes SG formation and innate antiviral immunity. To elucidate the action point of NSP5 in its inhibition of IFN expression, the promoter activity of the IFN-β luciferase reporter was stimulated using RIG-IN (the active form of RIG-I), MDA5, MAVS, TBK1, and IRF3-5D (the active form of IRF3), as well as STING and TRIF (the adaptor protein of the TLR3-IFN pathway). If NSP5 impairs IFN induction by any of these activators, it should act at or downstream of that activation point in the signaling pathway. The results showed that overexpression of NSP5 affected the activities of IFN-β-Luc (IFN-β luciferase reporter), IFN-λ1-Luc (IFN-λ1 luciferase reporter), and ISRE-Luc (ISG luciferase reporter) induced by RIG-IN and MDA5 but not MAVS, TBK1, IRF3-5D, TRIF, or STING (Fig. 4a). That is, NSP5 might antagonize RIG-I and MDA5. However, the SG component G3BP1 serves as a positive regulator of RLR receptors and functions upstream of RIG-I and MDA5. These phenomena revealed that NSP5 likely targeted multiple steps in the RLR pathway to inhibit the signaling cascade.

Since NSP5 attenuated avSG formation, we next examined whether NSP5 can interact with G3BP1, the hub protein of SGs. Results from fluorescence microscopy, co-IP, and in vitro pulldown assays indicated that NSP5 bound to G3BP1 (Fig. 4b-d; supplementary Fig. S2). Furthermore, NSP5 is a protease responsible for cleaving both cellular and viral proteins. 3C^pro^ of encephalomyocarditis virus was reported to cleave G3BP1 to attenuate SG formation.^17^ Therefore, we next investigated whether NSP5 can cleave G3BP1 to affect SG formation. We observed that NSP5 did not cleave G3BP1 (supplementary Fig. S3). In contrast, TAB1 was efficiently degraded by NSP5, congruent with previous findings (supplementary Fig. S3).^13^ To further validate whether the suppression of avSG formation is dependent on the cleavage of any avSG component, we generated an enzymatically inactive mutant, NSP5 C145A. We found that NSP5 C145A also suppressed SG formation, similar to wild-type NSP5 (Fig. 4e). These phenomena indicated that NSP5 attenuated avSG formation independent of its protease activity or cleavage of avSG components.

The subcellular localization of a protein is closely related to its biological function. Therefore, NSP5 localization was examined through confocal microscopy analysis. We transfected Myc-tagged NSP5 into HeLa cells and found that NSP5 was distributed in both the nucleus and cytoplasm (Fig. 4f), which is congruent with our previous study using Flag-tagged NSP5.^18^ We observed that NSP5 showed partial colocalization with the mitochondrial marker Tom20 but did not exhibit significant colocalization with the ER marker calnexin or the Golgi marker GM130 (Fig. 4f). We also investigated the relative localization of NSP5 with signaling molecules in the RLR signaling pathway, including RIG-I, MDA5, and MAVS. We observed that NSP5 did not colocalize with MAVS but showed obvious colocalization with RIG-I and MDA5 in immunofluorescence assays (Fig. 4f). Consistently, the co-IP and in vitro pulldown assays showed that NSP5 interacted with RIG-I and MDA5 rather than other signaling molecules (Figs. 4g, h; supplementary Fig. S2). More importantly, we found that NSP5 disrupted RIG-I–MAVS interaction, which is a crucial step in RIG-I signaling activation (Fig. 4i). This is consistent with the luciferase reporter assays showing that NSP5 inhibited the IFN activation induced by RIG-I but not MAVS (Fig. 4a). TRIM25, an E3 ubiquitin ligase, is essential for RIG-I-mediated antiviral activity. We found that NSP5 also impaired RIG-I–TRIM25 interaction (Fig. 4j), which is a crucial step in RIG-I activation. Therefore, NSP5 may target multiple steps of the RLR signaling pathway.

The phosphorylation of IRF3 is a critical step for IFN induction. Although RT-qPCR analysis and luciferase reporter assays indicated that NSP5 can suppress the induction of type I and type III IFNs (Fig. 3 and Fig. 4a), its effect on IRF3 phosphorylation during viral infection is unknown. We employed SeV as a substitute for SARS-CoV-2 to perform virus infection studies because of lacking a biosafety level 3 laboratory. To explore the effect of NSP5 on virus-induced IRF3 phosphorylation, normal HeLa cells and those expressing NSP5 were subjected to infection with SeV. We observed that there are more phosphorylated IRF3 in normal cells than that in NSP5-expressing cells (Fig. 4k). Consistent with our mechanistic study, we also observed that overexpression of NSP5 resulted in a reduced phosphorylation level of TBK1, which is a downstream event of RIG-I activation (Fig. 4k). The phosphorylation of IRF3 is a pivotal step upstream of its nuclear translocation to induce IFN expression. Because NSP5 inhibited IRF3 phosphorylation induced by SeV, we next investigated whether NSP5 affected IRF3 nuclear translocation. In resting cells, IRF3 was predominantly localized in cytosol regardless of the presence or absence of NSP5 (Fig. 4l), and SeV infection-induced IRF3 nuclear translocation in cells transfected with empty vector (Figs. 4l, m). However, SeV-induced IRF3 nuclear translocation was inhibited in cells expressing NSP5 compared with the corresponding control cells (Figs. 4l, m).

### SARS-CoV-2 N protein inhibits IFN induction by targeting G3BP1

**Fig. 5:**
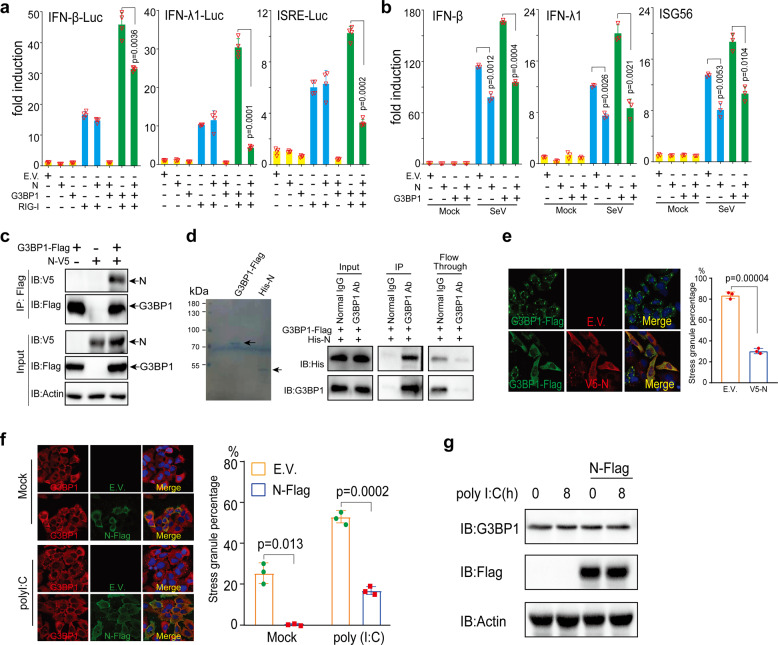
N protein inhibits G3BP1-mediated RIG-I signaling activation by suppressing SG formation. **a** and **b** N protein inhibits G3BP1-mediated RIG-I signaling activation. Thirty-six hours after transfection into HEK293T cells with indicated plasmids, the luciferase activities were measured using dual-luciferase assays (**a**). Twenty-four hours after transfection, HEK293T cells were infected with SeV (**b**), and 6 h after infection, the cells were harvested to determine the induction of IFN-β, IFN-λ1, and ISG56 using RT-qPCR. **c** and **d** N protein interacts with G3BP1. Co-IP analysis of the interaction between N-V5 and G3BP1-Flag in HEK293T cells (**c**). **d** Left panel: Coomassie blue staining analysis of the purified G3BP1-Flag and His-N proteins. Right panel: Co-IP analysis of the in vitro interaction between His-N and G3BP1-Flag. **e** N protein inhibits G3BP1-induced SG formation. Left panel: Confocal microscopic analysis of SG formation in HeLa cells transfected with G3BP1-Flag together with empty vector or expression vector of N for 24 h. Scale bars, 10 μm. Right panel: Quantification analysis of the percentage of SG formation (50 cells per sample). **f** N protein inhibits poly (I:C)-induced SG formation. Left panel: Confocal microscopic analysis of SG formation in HeLa cells transfected with empty vector or expression vector of N for 24 h followed by stimulation with poly (I:C) for 0 or 8 h. Scale bars, 10 μm. Right panel: Quantification analysis of the percentage of SG formation (200 cells per sample). **g** N protein does not affect G3BP1 expression. HeLa cells were transfected with plasmids as indicated, 16 h later, the cells were further stimulated with poly (I:C) by transfection. The expression of G3BP1, N-Flag, and actin was detected by immunoblotting. EV empty vector, h hours.

We next investigated the mechanism by which SARS-CoV-2 N protein counteracts SG formation and innate antiviral immunity. To elucidate the action point of N protein in its inhibition of IFN expression, the promoter activity of the IFN-β luciferase reporter was stimulated by RIG-IN, MDA5, MAVS, TBK1, and IRF3-5D, as well as STING and TRIF. We found that overexpression of N protein had no effect on the activities of IFN-β-Luc induced by RIG-IN, MDA5, MAVS, TBK1, IRF3-5D, TRIF, and STING. Similarly, N protein did not inhibit the activation of IFN-λ1-Luc or ISRE-Luc induced by the transfection of these signaling molecules (supplementary Fig. S4). This suggests that N protein might counteract an activator that functions upstream of RIG-I and MDA5. One such activator that promotes the activity of RIG-I and MDA5 is G3BP1, which is a positive regulator of RIG-I-mediated signaling and a probable cosensor of RLRs. We next examined whether the inhibitory effect of N protein on IFN expression is G3BP1 dependent. The inhibitory activity of N protein was examined in the presence or absence of G3BP1 and RIG-I. The results indicated that N protein suppressed the activities of IFN-β-Luc, IFN-λ1-Luc, and ISRE-Luc induced by RIG-I and G3BP1 but not RIG-I alone (Fig. 5a). Consistently, we observed that overexpression of G3BP1 enhanced the IFN-β, IFN-λ1, and ISG56 expression induced by SeV infection and partially reversed the inhibition of IFN-β by N protein (Fig. 5b). These evidences suggested that N protein antagonized the RLR signaling pathway by targeting upstream cofactors rather than directly affecting RLR receptors.

A previous proteomic study reported that N protein interacts with G3BP1.^19^ Therefore, we next studied the interaction between G3BP1 and N protein. The co-IP assays indicated that G3BP1 and N protein formed a complex in transfected HEK293T cells (Fig. 5c; supplementary Fig. S5). Furthermore, the purified G3BP1 protein coimmunoprecipitated with N protein in vitro (Fig. 5d), indicating a direct association between G3BP1 and N protein. When we performed a colocalization analysis of overexpressed G3BP1 and N protein, we observed that N protein overexpression substantially decreased SG formation induced by overexpression of G3BP1 (Fig. 5e). The percentage of cells with SG decreased from 84 to 30% (Fig. 5e), suggesting that N protein inhibits the spontaneous SG formation caused by G3BP1 overexpression. Since SGs are an essential platform in RLR signal transduction, we examined whether N protein also negatively regulates SG formation caused by various types of nucleic acid transfection. Consistent with the screening results, we found that transfection of the control vector plasmid led to SG formation in approximately 25% of the cells, while transfection of a plasmid expressing Flag-tagged N protein almost completely abolished SG formation (Fig. 5f). Previous studies also suggested that poly (I:C) can trigger the formation of avSG.^6^ Congruently, we observed that poly (I:C) stimulation further increased the percentage of cells with SGs from 25 to 53%, while overexpression of N protein decreased this percentage to approximately 17% (Fig. 5f). We observed that overexpression of N protein did not affect the endogenous G3BP1 level with or without poly (I:C) transfection, suggesting that N protein modulated SG formation independent of G3BP1 cleavage (Fig. 5g).

### SARS-CoV-2 N protein suppresses PACT-induced RLR signaling activation

**Fig. 6:**
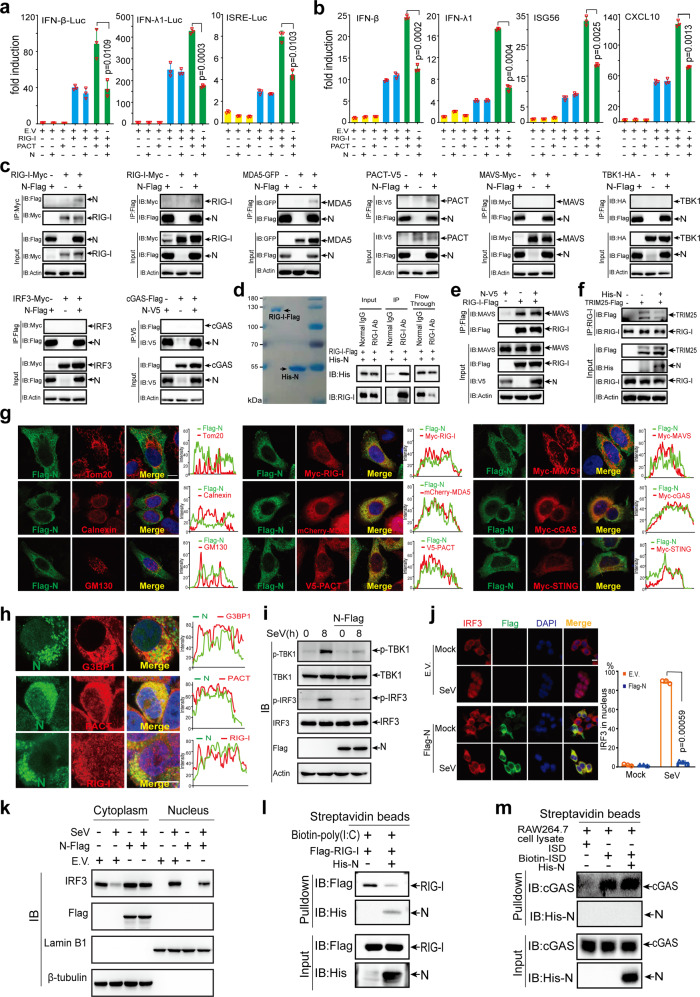
N protein prevents PACT-induced RIG-I signaling activation. **a** and **b** N protein inhibits PACT-mediated RIG-I signaling activation. IFN-β, IFN-λ1, or ISRE luciferase reporters and protein-expressing plasmids were transfected into HEK293T cells as indicated. Thirty-six hours after transfection (**a**), dual-luciferase assay was used to measure the luciferase activities. Forty-eight hours after transfection (**b**), the cells were harvested to analyze the expression of IFN-β, IFN-λ1, ISG56, and CXCL10 using RT-qPCR. **c** The N protein interacts with RIG-I, MDA5, and PACT rather than with MAVS, TBK1, IRF3, or cGAS. Protein expression plasmids were transfected into HEK293T cells as indicated, 24 h later, the cells were lysed for co-IP. **d** N protein directly binds to RIG-I. Left panel: Coomassie blue staining analysis of the purified RIG-I-Flag and His-N proteins. Right panel: Co-IP analysis of the in vitro interaction between RIG-I-Flag and His-N. **e** N protein does not affect RIG-I–MAVS interaction using co-IP assays in HEK293T cells. **f** N protein disrupts RIG-I–TRIM25 interaction. HEK293T cells were subjected to transfection with the protein expression plasmids as indicated, 24 h later, the cells were lysed for co-IP. **g** Representative confocal images of N protein with the indicated organelles or signaling molecules in HeLa cells. Scale bar, 10 µm. **h** Colocalization between endogenous N protein and signaling molecules. HEK293T cells were transfected with pBAC-nCoV-Replicon plasmid to obtain the endogenous expression of viral proteins for 24 h. Immunofluorescence staining was performed with antibodies as indicated. **i** N protein inhibits the phosphorylation of TBK1 and IRF3 induced by SeV infection. HeLa cells were transfected with empty vector or N protein-expressing plasmids; 16 h later, the cells were subsequently infected with SeV as indicated. The protein levels of total TBK1 and total IRF3 as well as phosphorylated (p-) TBK1, p-IRF3, and N protein were detected by immunoblotting. **j** and **k** N protein blocks SeV-induced IRF3 nuclear translocation. **j** Left panel: Confocal microscopic analysis of IRF3 localization in HEK293T cells transfected with empty vector or expression vector of N protein for 24 h, followed by SeV infection as indicated. Scale bars, 10 μm. Right panel: Quantification analysis of IRF3 nuclear localization (50 cells per sample). **k** IRF3 expression in the cytoplasmic and nuclear fractions. HEK293T cells were transfected with an empty vector or expression vector of N protein for 24 h followed by SeV infection for 6 h. Afterward, the cells were harvested and separated into cytoplasmic and nuclear portions. Each portion was analyzed by immunoblot for the detection of IRF3, N protein, Lamin B1 (nuclear marker), and β-tubulin (cytoplasmic marker). **l** N protein prevents poly (I:C) binding to RIG-I. Pulldown analysis of Flag-RIG-I from transfected HEK293T cells binding to poly (I:C) with or without N protein. **m** N protein does not affect the association between ISD and cGAS. Pulldown analysis of cGAS from RAW264.7 cells binding to ISD with or without the N protein. EV empty vector, h hours.

SG serves as an indispensable platform for RLR recognition of nucleic acids. Multiple RLR receptors and positive regulators of RLRs are localized in SGs to exert their antiviral innate immunity role.^20^ Since the SARS-CoV-2 N protein targets cofactors upstream of RLR receptors and modulates SGs, we investigated whether N protein also attenuates the positive regulation of RLRs by other cofactors. One such cofactor is PACT, which is critical for the recognition of dsRNA by RLR receptors.^21^ The inhibitory activity of N protein was examined in the presence or absence of PACT and RIG-I. We found that N protein inhibited the activities of IFN-β-Luc, IFN-λ1-Luc, and ISRE-Luc induced by RIG-I and PACT but not by RIG-I alone (Fig. 6a). Consistently, we observed that N protein inhibited the induction of IFN-β, IFN-λ1, ISG56, and CXCL10 by RIG-I and PACT but not by RIG-I alone (Fig. 6b). These results suggested that N protein affects a series of RLR cofactors to inhibit type I and type III IFN expression by modulating avSG formation.

Furthermore, we observed that N protein interacted with PACT, RIG-I, and MDA5 but not with other signaling molecules (Fig. 6c). Although N protein directly binds to RIG-I, it cannot affect RIG-I–MAVS interaction (Figs. 6d, e). However, overexpression of N protein impaired RIG-I–TRIM25 interaction (Fig. 6f). We also observed that N protein colocalized with PACT, RIG-I, and MDA5 rather than with MAVS or STING through confocal microscopy analysis (Fig. 6g). When N protein was expressed by transfection of pBAC-nCoV-Replicon plasmid (supplementary Fig. S6), it colocalized with endogenous G3BP1, PACT, and RIG-I (Fig. 6h). As mentioned above, the phosphorylation of IRF3 is essential for type I and type III IFN activation. We next studied whether N protein affects IRF3 phosphorylation during SeV infection. To explore the effect of N protein on IRF3 phosphorylation induced by viral infection, HeLa cells and HeLa cells expressing N protein were subjected to infection with SeV. We observed that SeV infection could trigger robust phosphorylation of IRF3 in HeLa cells, while the phosphorylation of IRF3 was significantly attenuated in HeLa cells expressing N protein (Fig. 6i). Consistent with our mechanistic study, we also observed that overexpression of N protein resulted in a reduced phosphorylation level of TBK1 (Fig. 6i). Moreover, we observed that the nuclear translocation of IRF3 triggered by SeV infection was prevented in cells transfected with the plasmid of N protein compared with cells transfected with empty vector (Fig. 6j, k).

N protein harbors NTD and CTD, which bind to the viral RNA genome. Therefore, we hypothesize that N protein likely affects the recognition of dsRNA by RIG-I. To test this hypothesis, we incubated Flag-RIG-I with biotin-labeled poly (I:C) with or without the addition of N protein. We observed that N protein significantly inhibited the binding of poly (I:C) to RIG-I (Fig. 6l). As a control, N protein did not affect the binding of dsDNA to cGAS (Fig. 6m).

## Discussion

In this study, we systematically investigated the possible SASRS-CoV-2 proteins involved in regulating avSG formation caused by nucleic acid transfection. Through a comprehensive screening, we observed that NSP5 and N protein strongly inhibited avSG formation. Given the close association between avSG and the IFN response, we then examined the roles of NSP5 and N protein in innate antiviral immunity and found that both inhibited the expression of type I and type III IFNs as well as the downstream ISGs induced by SeV infection or poly (I:C) transfection. In addition, NSP5 and N protein also inhibited the phosphorylation of TBK1 and IRF3, as well as the nuclear translocation of IRF3 induced by SeV infection, suggesting that NSP5 and N protein suppressed downstream RLR signaling. Mechanistically, we found that NSP5 and N protein utilized different strategies to impair innate antiviral immunity, even though both suppressed avSG. N protein interacts with G3BP1 to sequester G3BP1 from forming SGs, prevents cofactors of RLRs from activating RLR, and impairs the recognition of dsRNA by RIG-I. NPS5 not only affects SG formation but also impedes the interaction between RLR and MAVS to suppress downstream signaling. N protein and NSP5 are not the only proteins that can counteract the antiviral response in two ways, suppressing both the SG formation and innate immunity. 2A^pro^ of the enterovirus and p4a of MERS-CoV also antagonize both antiviral responses,^7,22,23^ again suggesting that these two antiviral pathways are tightly associated with each other.

A recent study suggested that SARS-CoV-2 NSP5 antagonizes type I IFN production and affects JAK-STAT signaling.^24^ Similar to our findings, they also found that NSP5 impairs the activation of IFN by RIG-IN. However, they proposed that SARS-CoV-2 NSP5 affects TRIM25-mediated RIG-I ubiquitination. TRIM25 is a cofactor upstream of RIG-I and is recruited to avSG,^20,25^ and our study found that NSP5 disrupted the avSG formation and the interaction between RIG-I and MAVS. Therefore, it is most likely that NSP5 targets both upstream and downstream RLRs to affect the IFN response. We also discovered that NSP5 affected avSG formation independently of its protease activity and did not cleave G3BP1. In contrast, NSP5 efficiently degrades TAB1, which was recently found to be a protease substrate of NSP5.^13^ Therefore, NSP5 modulates the host cell machinery in both protease-dependent and protease-independent manners.

A previous study suggested that the N protein of SARS-CoV interacts with TRIM25 and impedes the K63-linked polyubiquitination of RIG-I to suppress type I IFN production.^26^ Similar to SARS-CoV N protein, the N protein of MERS-CoV also inhibits type I and type III IFN production by impairing the TRIM25-mediated K63-linked polyubiquitination of RIG-I.^27^ The N protein of porcine delta coronavirus (PDCoV) also utilizes a similar strategy to affect the ubiquitination of RIG-I to inhibit the IFN response.^28^ In this study, we found that the N protein of SARS-CoV-2 specifically targets upstream of RIG-I, disrupts avSG formation, and prevents the cofactors G3BP1 and PACT from enhancing the activity of RIG-I. Given the similarity of the peptide sequences of N proteins within these viruses, it would be interesting to examine whether the N proteins from SARS-CoV, MERS-CoV, and PDCoV also modulate SG formation.

Although we found that SARS-CoV-2 is equipped with N protein and NSP5 to modulate the stress response, it is urgent to determine whether SARS-CoV-2 infection stimulates or inhibits SG formation. A possible scenario is that SARS-CoV-2 can suppress avSG formation through N protein and NSP5 and adopt certain components of SGs to form atypical SGs to facilitate viral replication. A recent study also reported that the N protein of SARS-CoV-2 blocks SG formation caused by sodium arsenite.^29^ In our case, we found that the SARS-CoV-2 N protein blocked SG formation caused by nucleic acids, which is a different biogenesis pathway from oxidative stress. These phenomena suggest that the SARS-CoV-2 N protein most likely targets downstream hub proteins of SG, such as G3BP1, instead of upstream kinases for EIF2α phosphorylation to dampen SG formation. The exact mechanism by which the SARS-CoV-2 N protein affects SG formation requires further investigation.

A previous study showed that treatment of cells with an mRNA translation inhibitor, which enhances the aggregation of SG, inhibited SARS-CoV-2 infection in vitro, suggesting that SG is an important innate defense system and may be a novel drug target for treating COVID-19.^19,30^ This is consistent with the phenomenon that SARS-CoV-2 N protein and NSP5 facilitated VSV infection. However, N protein and NSP5 modulated this process through two mechanisms: perturbation of SG formation and suppression of the IFN response. Interestingly, our previous studies also observed that the M protein and ORF9b of SARS-CoV-2 attenuated the IFN response.^31,32^ However, neither inhibited SG formation in our screening. These phenomena suggest that SARS-CoV-2 is equipped with multiple viral proteins that can target the various steps of the IFN signaling pathway involved in host antiviral defense.

## Materials and methods

### Cell culture and transfection

HeLa, HEK293T, and Vero cells were grown under standard conditions in Dulbecco’s modified Eagle’s medium (DMEM, Gibco, USA) with the addition of 10% (v/v) fetal bovine serum (FBS, Gibco, USA), 100 U/mL penicillin, and 100 μg/mL streptomycin. A549 cells, a human lung carcinoma epithelial cell line, were maintained in Ham’s F-12K medium (Gibco, USA) containing 10% FBS. All cells were cultured at 37 °C in a humidified incubator with 5% CO_2_. Polyethylenimine ‘Max’ (Polysciences, Inc., Germany) was used to transfect plasmids into HEK293T cells, and Lipofectamine 3000 (Invitrogen, USA) was used to transfect plasmids into A549 and HeLa cells. Poly (I:C) (Sigma P1530, USA) was transfected into cells using Lipofectamine 2000 (Invitrogen, USA) as described previously.^33^

### Plasmids

PACT, RIG-I, RIG-IN, MDA5, MAVS, TBK1, IKKε, IRF3-5D, TRIF, and STING plasmids were constructed as described previously.^34–36^ The IFN-β luciferase reporter pGL3-IFN-β-Luc, IFN-λ1 luciferase reporter pGL3-IFN-λ1-Luc, and ISG luciferase reporter pISRE-Luc were described in our previous studies.^37,38^ SARS-CoV-2 N (NCBI access No. YP_009724397) and NSP5 protein genes (NCBI access No. YP_009725301) were cloned into the pCAG vector. pENTER-G3BP1-Flag was purchased from Vigene Biosciences, China. Plasmids expressing the domain deletion mutants of SARS-CoV-2 NSP5 were constructed according to a previous publication.^39^ The pBAC-nCoV-Replicon plasmid with deletion of the *S* gene which can express the subgenomic RNAs similar to the profiles of the wild-type SARS-CoV-2 was provided by Prof. Ji-An Pan and Prof. Deyin Guo.^40^ Plasmids expressing the domain deletion mutants of SARS-CoV-2 N protein including △NTD, △SR, and △CTD were provided by Prof. Jun Cui.^41^ The sequences of the primers used in this study are provided in supplementary Table 1.

### Antibodies and reagents

The antibodies used were mouse anti-Flag M2 antibody from Sigma-Aldrich (USA); rabbit anti-Flag tag (D6W5B), rabbit anti-RIG-I (D14G6), rabbit anti-IRF3 (D83B9), rabbit anti-pIRF3 (4D46), rabbit anti-TBK1 (3031 S), and rabbit anti-pTBK1 (D52C2) from Cell Signaling Technology (USA); rabbit anti-calnexin, rabbit anti-SARS-CoV-2 N, mouse anti-actin, and rabbit anti-G3BP1 antibodies from Proteintech (China); mouse anti-Myc (9E10) antibody from Origene (USA); rabbit anti-TAB1 and rabbit anti-GM130 antibodies from Abcam (United Kingdom); mouse anti-GAPDH antibody (AF0006) from Beyotime (China); rabbit anti-Lamin B1, mouse anti-SARS-CoV-2 N, and mouse anti-β-Tubulin (C66) antibodies from Abmart (China); mouse anti-PACT (D-4) and mouse anti-G3BP1 (H-10) antibodies from Santa Cruz Biotechnology (USA); rabbit anti-IRF3 (CY5779) and rabbit anti-IRF3 (CY6575) antibodies from Abways (China); mouse anti-HA antibody from MDL Biotech (China); Alexa Fluor 488 goat anti-rabbit IgG, Alexa Fluor 568 goat anti-mouse IgG, Alexa Fluor 488 goat anti-mouse IgG, and Alexa Fluor 568 goat anti-rabbit IgG secondary antibodies from Invitrogen (USA). Protein A/G beads were purchased from Santa Cruz Biotechnology (USA), and anti-Flag or anti-Myc magnetic beads were purchased from Bimake (USA). Interferon stimulatory DNA (ISD) and biotin-labeled ISD were synthesized by Sangon Biotech (China). Poly (I:C) and biotin-labeled poly (I:C) were obtained from Invivogen (USA).

### Real-time quantitative PCR

Total cellular RNAs were isolated using TRIzol Reagent (Invitrogen, USA) and reverse-transcribed into first-strand cDNA with a HiScript III 1st Strand cDNA Synthesis Kit (Vazyme, China). Real-time quantitative PCR (RT-qPCR) was conducted using UltraSYBR Mixture (CWBIO, China) and a LightCycler 96 instrument (Roche) with the expression of GAPDH as the internal control as described.^36,42^

### Luciferase reporter assays

The dual-luciferase reporter assay system was conducted to determine the relative activity of luciferase reporters as described in our previous studies.^36,42^ HEK293T cells were transfected with the luciferase reporter plasmids and the protein expression plasmids as indicated in each experiment. After transfection for 36 h, the cells were lysed for measurement of luciferase activity using a Dual Luciferase Reporter Assay Kit (Vazyme, China).

### Viruses and infection

HeLa or HEK293T cells were infected with VSV-eGFP or SeV as described previously.^34–36^ Briefly, cells were first washed with prewarmed serum-free DMEM at 37 °C followed by infection with the viruses at the desired multiplicity of infection (MOI). After incubation with the viruses for 1 h, the supernatant was discarded, and fresh DMEM with FBS was replenished.

### Coimmunoprecipitation and immunoblotting

For the coimmunoprecipitation (co-IP) assay, HEK293T cells were harvested 24 h after transfection and lysed in ice-cold lysis buffer (50 mM Tris-HCl pH 7.4, 50 mM EDTA, 1.0% NP-40, and 150 mM NaCl) containing 1 × protease and phosphatase inhibitor cocktail from Sigma as described previously.^34,35^ Following centrifugation at 14,000 g for 10 min at 4 °C to remove cell debris, the protein concentration was measured using the bicinchoninic acid assay (Pierce, USA) and co-IP was performed with the indicated antibodies and beads for overnight at 4 °C. Afterward, beads were washed with lysis buffer for four times before boiling with 2×SDS loading buffer [0.1 M Tris-HCl pH 6.8, 4% (w/v) SDS, 20% (v/v) glycerol, 0.2% (w/v) bromophenol blue, and 1% (v/v) 2-mercaptoethanol] to elute the immunoprecipitates.

For immunoblotting, cells were lysed by the M-PER Protein Extraction Reagent (Pierce, USA) supplemented with 1 × protease and phosphatase inhibitor cocktail. The cytoplasmic and nuclear extracts were fractionated with a Nuclear and Cytoplasmic Protein Extraction Kit (Beyotime, China). Equal amounts of proteins from different samples were loaded on the SDS-PAGE gels. The proteins on SDS-PAGE gels were then transferred onto PVDF membranes (Millipore). After being blocked with 5% fat-free milk, the membranes were incubated with specific primary antibodies at 4 °C overnight, followed by incubating with HRP-conjugated secondary antibodies at 25 °C for 1 h. The specific proteins were visualized with ECL Western blotting detection reagent (Pierce, USA) as described previously.^31^

### Immunofluorescence

Confocal immunofluorescence microscopy studies were conducted as described previously.^31^ In brief, 5 × 10^4^ HeLa cells or 1 × 10^5^ HEK293T were seeded into 12-well slides 24 h before transfection or infection. Following transfection or infection, the cells were then subjected to the fixation, permeabilization, and blocking with reagents from an immunofluorescence assay kit (Beyotime, China). The cells were subsequently reacted with the indicated primary antibodies overnight at 4 °C and fluorescent secondary antibodies for 1 h at room temperature. The slides were mounted with mounting medium with DAPI (Abcam, USA). Fluorescence images were captured and analyzed using a Zeiss LSM880 confocal microscope.

### Poly (I:C) or ISD pulldown assay

HEK293T cells cultured in 6 cm dishes were transfected with 6 µg plasmids expressing Flag-RIG-I for 24 h. The cells were lysed with lysis buffer (0.15 M NaCl, 1% NP40, 0.05 M Tris pH = 7.4) supplemented with 1 × protease and phosphatase inhibitor cocktail. After centrifugation for 10 min at 14,000 g, supernatants with or without the addition of His-N protein were added with 2 µg poly (I:C) or 2 µg biotin-labeled poly (I:C) (Invivogen, USA) for 2 h. Afterward, the mixture was pulled down by adding streptavidin beads for another 2 h followed by washing with washing buffer (0.3 M NaCl, 1% NP40, 0.05 M Tris pH = 7.4) 3 times and elution by adding 2×SDS loading buffer. For ISD pulldown, the same procedures were performed. ISD and biotin-labeled ISD were synthesized by Sangon Biotech (China), and 2 µg of each was mixed with the cell lysates.

### Statistics

Results are presented as the mean ± SEM and represent one of three independent experiments. Statistical analysis was performed using GraphPad Prism 8.0 and Microsoft Excel. The two-tailed unpaired Student’s *t*-test was conducted to determine the significance. The p values are marked within each figure or figure legend. The p values less than 0.05 were considered statistically significant.

## Supplementary information

Ms SIGTRANS-03912R1 Supplementary Materials

## Data Availability

All data and materials are available to the researchers once published.
