# Supplementary material for: SARS-CoV-2 NSP5 and N protein counteract the RIG-I signaling pathway by suppressing the formation of stress granules
Source: Signal Transduct Target Ther. 2022 Jan 24;7:22. doi: 10.1038/s41392-022-00878-3 (PMC8785035; doi:10.1038/s41392-022-00878-3)
Supplement: Supplementary file 1 — Ms SIGTRANS-03912R1 Supplementary Materials [file 41392_2022_878_MOESM1_ESM.docx]

Supplementary Materials for

**SARS-CoV-2 NSP5 and N Protein Counteract the RIG-I Signaling Pathway by Suppressing the Formation of Stress Granules**

Yi Zheng ^1^, Jian Deng ^2^, Lulu Han ^1^, Meng-Wei Zhuang ^2^, Yanwei Xu ^1^, Jing Zhang ^2^, Mei-Ling Nan ^2^, Yang Xiao ^2^, Peng Zhan ^3, 4^, Xinyong Liu ^3, 4^, Chengjiang Gao ^1,^ ^*^, Pei-Hui Wang ^2, 5,^ ^*^

^1^ Key Laboratory of Infection and Immunity of Shandong Province, Department of Immunology, School of Basic Medical Sciences, Cheeloo College of Medicine, Shandong University, Jinan 250012, China

^2^ Key Laboratory for Experimental Teratology of Ministry of Education and Advanced Medical Research Institute, Cheeloo College of Medicine, Shandong University, Jinan 250012, China

^3^ Department of Medicinal Chemistry, Key Laboratory of Chemical Biology (Ministry of Education), School of Pharmaceutical Sciences, Cheeloo College of Medicine, Shandong University, 44 West Culture Road, 250012, Jinan, Shandong, PR China

^4^ China–Belgium Collaborative Research Center for Innovative Antiviral Drugs of Shandong Province, 44 West Culture Road, 250012, Jinan, Shandong, PR China

^5^ Suzhou Research Institute, Shandong University, Shandong University, Suzhou, Jiangsu 215123, China

*Correspondence to: Chengjiang Gao ([cgao@sdu.edu.cn](mailto:cgao@sdu.edu.cn)) or Pei-Hui Wang ([pei-hui.wang@connect.hku.hk](mailto:pei-hui.wang@connect.hku.hk))

These authors contributed equally: Yi Zheng, Jian Deng, Lulu Han

**This PDF file includes:**

Figures S1 to S6

Tables S1


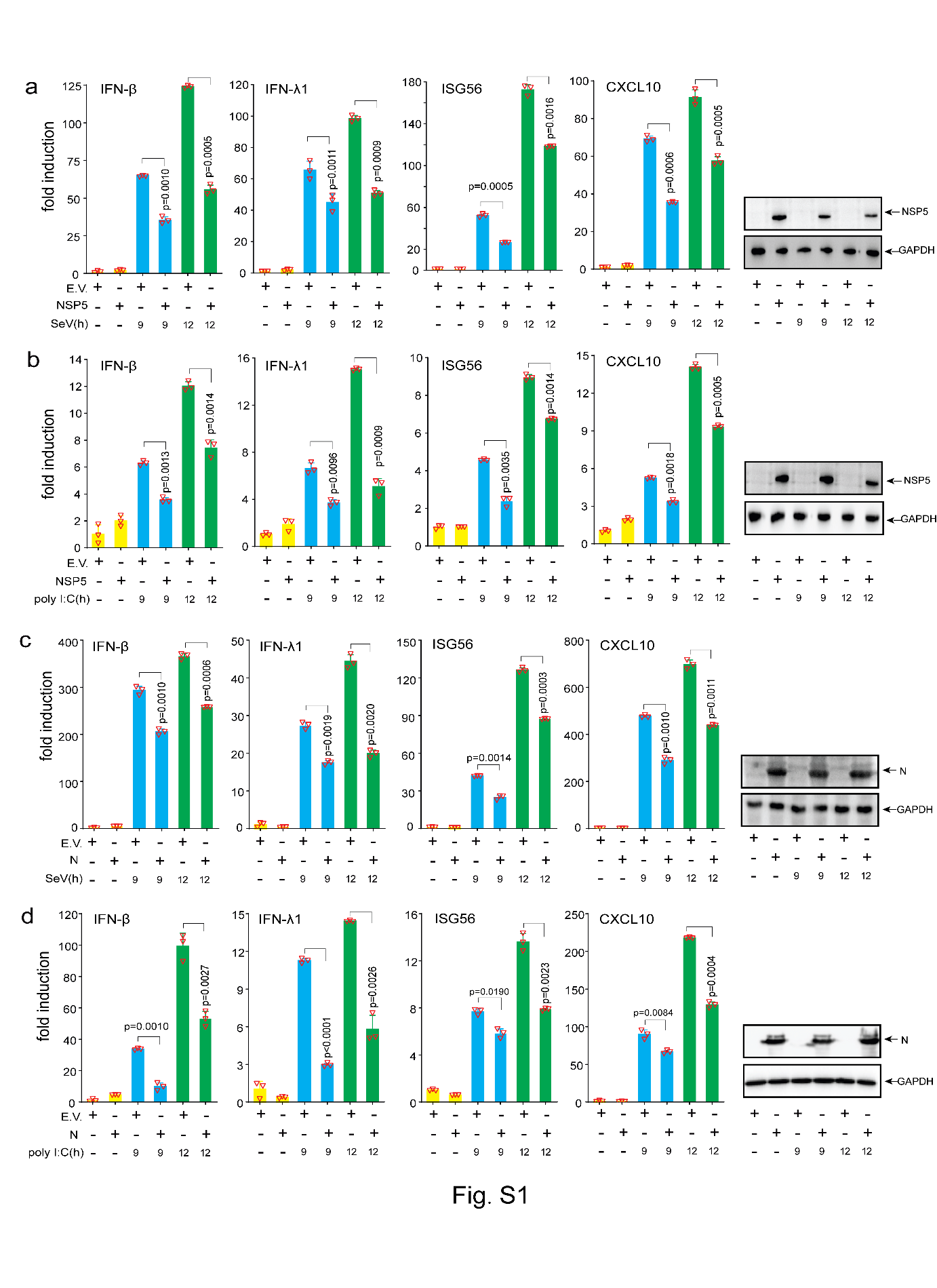


Figure. S1.

**NSP5 and N protein inhibit IFN production in HEK293T cells.** Plasmids were transfected into HEK293T cells as indicated. Twenty-four hours later, the cells were stimulated by SeV infection (**a** and **c**) or poly (I:C) transfection (**b** and **d**). At 9 and 12  hours after stimulation, the cells were harvested for RNA extraction and subsequent RT-qPCR analysis. Three independent biological replicates were analyzed, and one representative is shown. The error bars indicate the SEM values of technical triplicates.. Statistical significance is shown as indicated.


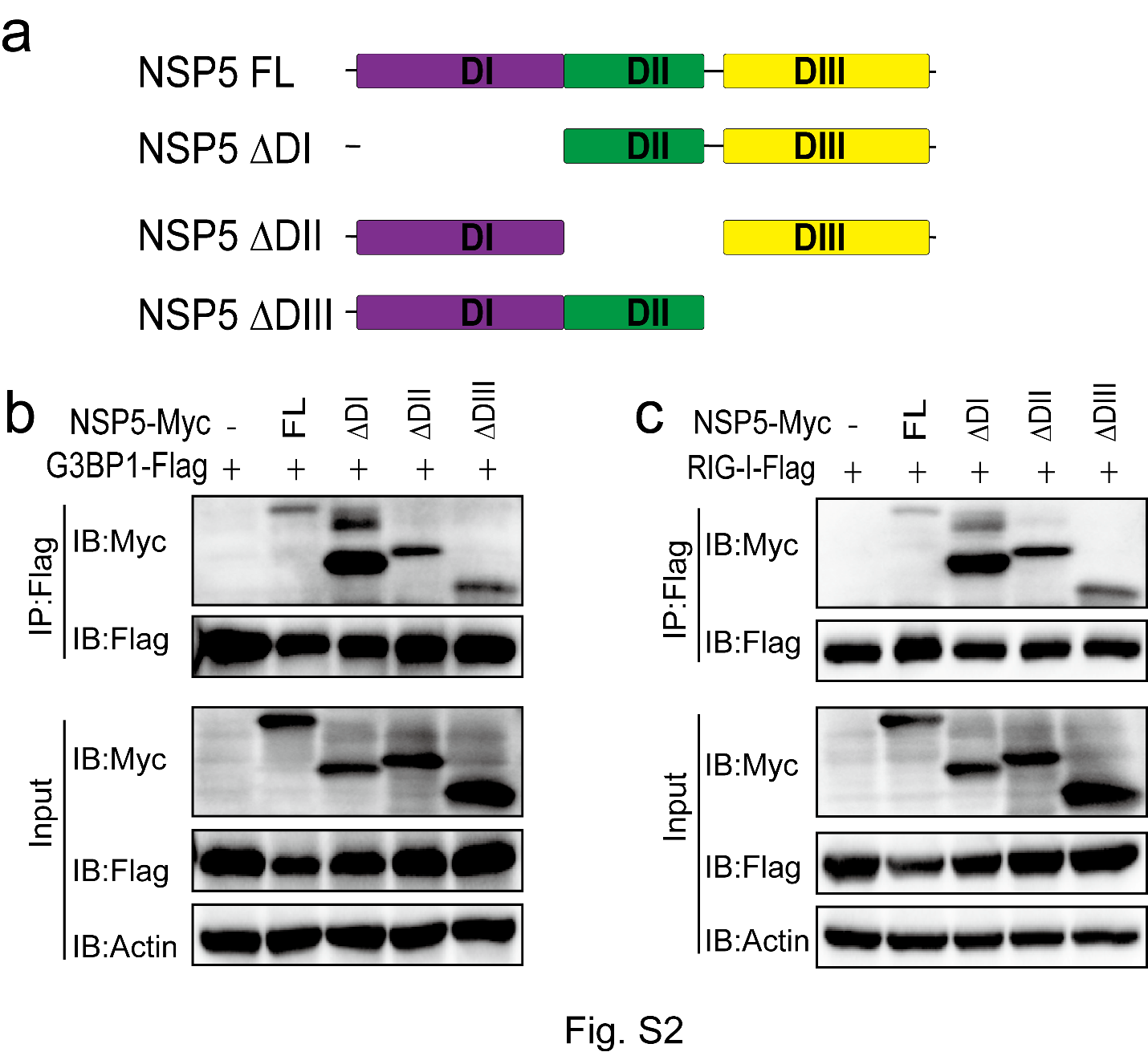


Figure. S2.

**Domain mapping of the NSP5-G3BP1 and NSP5**–**RIG-I interaction. a** Domain organization of SARS-CoV-2 NSP5 and its domain deletion mutants. **b** and **c** The interaction of G3BP1 and RIG-I with NSP5 mutants. HEK293T cells were transfected with the indicated plasmids for 24 hours before coimmunoprecipitation with the anti-Flag magnetic beads. The input and immunoprecipitates were immunoblotted with the indicated antibodies. Full-length, FL; Domain I, DI; Domain II, DII; Domain III, DI.


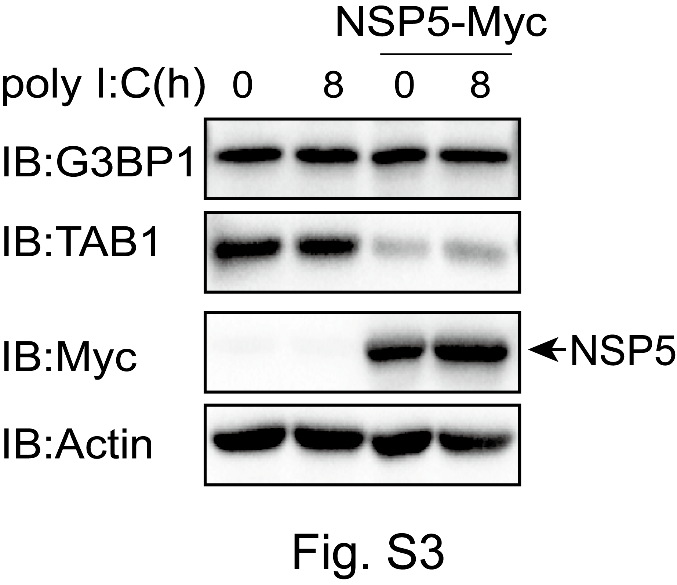


Figure. S3.

**Overexpression of NSP5 causes the degradation of TAB1 but not G3BP1.** HeLa cells were transfected with an empty vector or NSP5-expressing plasmid; 16 hours later, the cells were stimulated by poly (I:C) transfection for the indicated time. The expression of G3BP1, TAB1, and actin was analyzed using immunoblotting.


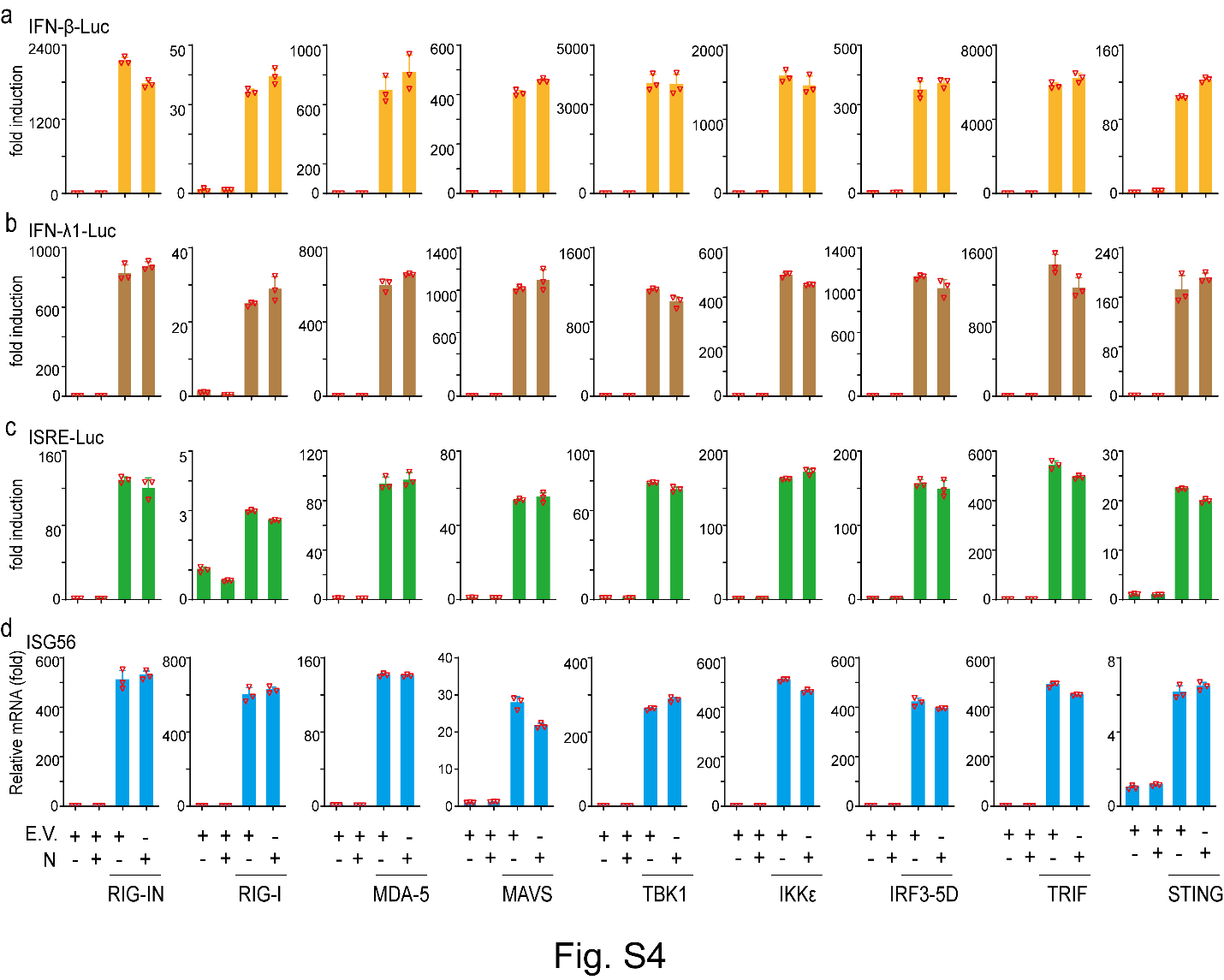


Figure. S4.

**SARS-CoV-2 N protein did not affect the activities of IFN production induced by RIG-IN, MDA-5, MAVS, TBK1, IRF3-5D, TRIF, or STING.** The empty vector pcDNA6B (E.V., 500 ng) or SARS-CoV-2 N protein-expressing plasmid (500 ng) and luciferase reporter plasmids (**a**-**c**) were transfected into HEK293T cells together with the protein-exressing plasmids as indicaded. Thirty-six hours after transfection, the cells were harvested and the luciferase activities were evaluated by dual luciferase assays. **d** HEK293T cells were transfected with plasmid as indicaded. Thirty-six hours later, cells were harvested for RT-qPCR analysis. Three independent biological replicates were analyzed, the results of one representative experiment are shown, and the error bars indicate the SEM values.


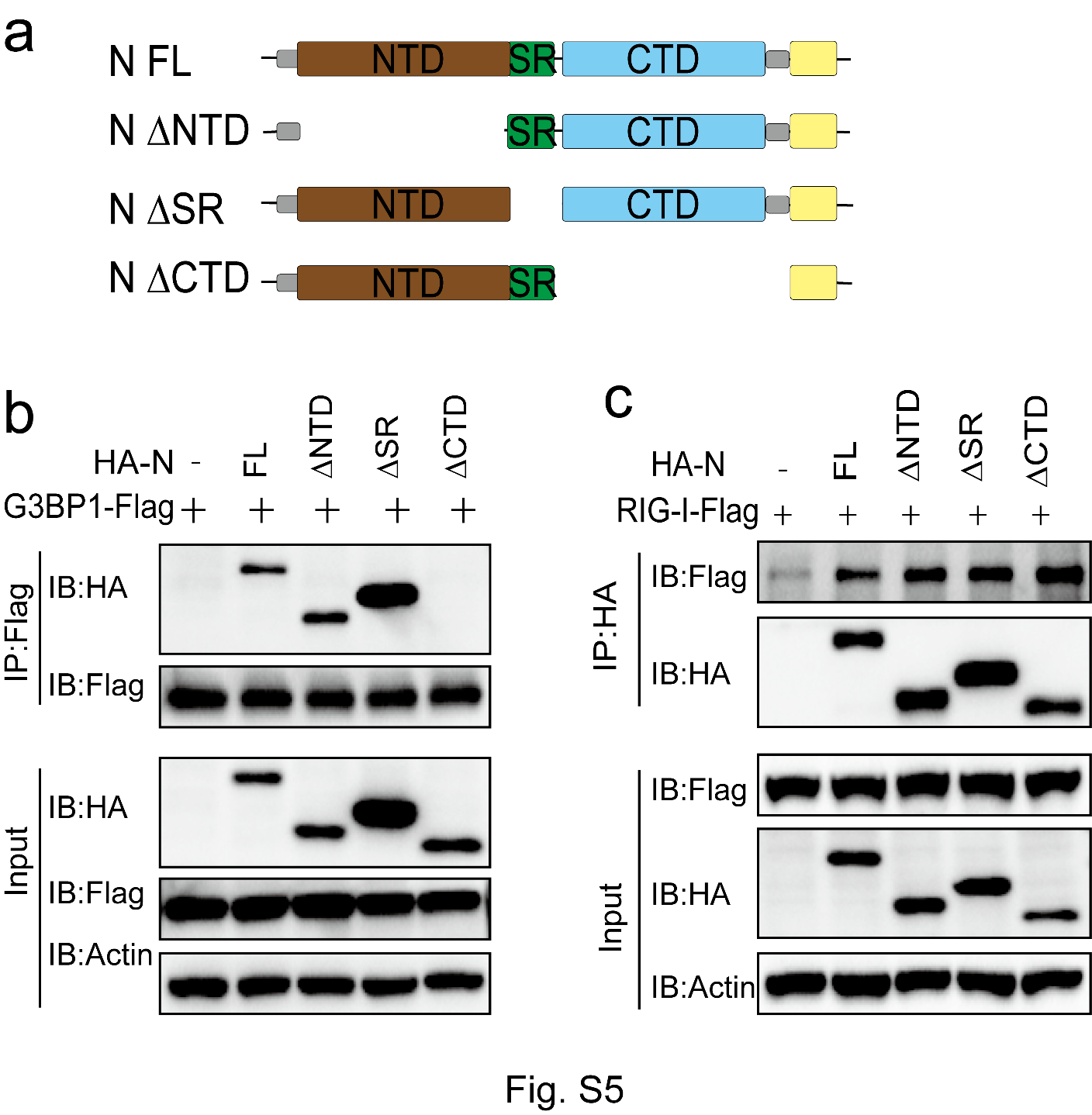


Figure. S5.

**Domain mapping of the N-G3BP1 and N-RIG-I interaction. a** Domain organization of SARS-CoV-2 N protein and its domain deletion mutants. **b** and **c** The interaction of G3BP1 and RIG-I with SARS-CoV-2 N protein mutants. Plasmids were tranfected into HEK293T cells as indicated for 24 hours before coimmunoprecipitation with the anti-Flag or anti-HA magnetic beads. The input and immunoprecipitates were immunoblotted with the indicated antibodies. Full-length, FL; N-terminal domain, NTD; serine- and arginine-rich tract; SR; C-terminal domain, CTD


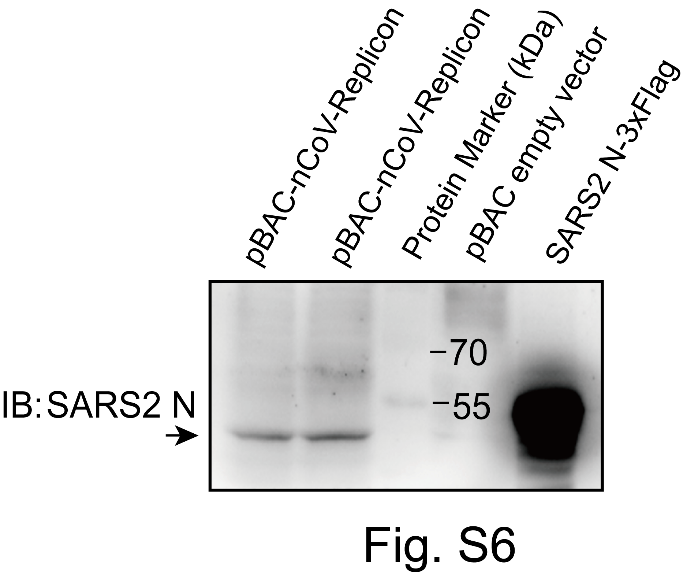


Figure. S6.

**The expression of SARS-CoV-2 N protein by the pBAC-nCoV-Replicon.** HEK293T cells cultured in 12-well plates (4 × 10^5^ cells per well) were transfected with pBAC empty vector, pBAC-nCoV-Replicon plasmid, and N protein-expressing plasmid. Forty-eight hours after transfection, the cells were harvested for immunoblot analysis with antibody against SARS-CoV-2 N protein. Immunoblotting results are representative of two experiments with similar results. SARS-CoV-2, SARS2

Table S1.

Primers used in this study.

| Primer name | Sequence (5′-3′) | Usage |
| --- | --- | --- |
| GAPDH-F  GAPDH-R | GGAGCGAGATCCCTCCAAAAT  GGCTGTTGTCATACTTCTCATGG | RT-qPCR |
| IFN-β-F  IFN-β-R | TTGCTCTCCTGTTGTGCTTC  AAGCCTCCCATTCAATTGCC | RT-qPCR |
| IFN-λ1-F  IFN-λ1-R | GAGGCCCCCAAAAAGGAGTC  AGGTTCCCATCGGCCACATA | RT-qPCR |
| ISG56-F  ISG56-R | CTAAGCAAAACCCTGCAGAAC  TCAGGCATTTCATCGTCATC | RT-qPCR |
| CXCL10-F  CXCL10-R | GTGGCATTCAAGGAGTACCTC  GACCTTTCCTTGCTAACTGCT | RT-qPCR |
| N-F  N-R | GCACAGTGGCGGCCGCTCGAGGCCACCATGAGTGGTTTTAGAAAAATGGCATTC  GTCATCCTTGTAATCTCTAGATTGGAAAGTAACACCTGAGCATT | expression plasmid |
| NSP5-F  NSP5-R | GCACAGTGGCGGCCGCTCGAGGCCACCATGTCTGATAATGGACCC  GTCATCCTTGTAATCTCTAGAGGCCTGAGTTGAGTCAGCA | expression plasmid |
|  |  |  |
| △DI-F  △DI-R | CTATAGGGCAAGCTTGGTACCGCCACCATGTCTGGTTTTAGGAAAATGGCGAAATTCGTCCGAATCCAACCAG  GATGAGTTTTTGTTCTCTAGACTGGAATGTCACGCCGGAG | expression plasmid |
|  |  |  |
| △DII-F  △DII-R | CTATAGGGCAAGCTTGGTACCGCCACCATGTCTGGTTTTAGGAAAATGGC  AAGACGCCCAAGTATTTCGTTGACCGGCAAACC | expression plasmid |
|  |  |  |
| NSP5-F  NSP5-R | CTATAGGGCAAGCTTGGTACCGCCACCATGTCTGGTTTTAGGAAAATGGC  GATGAGTTTTTGTTCTCTAGAAATCGTTGTGTCGGTTCCAGC | expression plasmid |

F: forward primer. R: reverse primer.
